# Bruxism Frequency and Low-Intensity Occlusal Tactile Detection in Healthy Adults

**DOI:** 10.3390/jcm15093469

**Published:** 2026-05-01

**Authors:** Marko Zlendić, Ema Vrbanović Đuričić, Iva Biloš, Ivan Boras, Ivan Alajbeg, Iva Z. Alajbeg

**Affiliations:** 1 Department of Removable Prosthodontics, University of Zagreb School of Dental Medicine, 10000 Zagreb, Croatia; mzlendic@sfzg.unizg.hr (M.Z.); evrbanovic@sfzg.unizg.hr (E.V.Đ.); 2 University of Zagreb School of Dental Medicine, 10000 Zagreb, Croatia; bilosavi@gmail.com (I.B.); iboras@sfzg.hr (I.B.); 3 Department of Oral Medicine, University of Zagreb School of Dental Medicine, 10000 Zagreb, Croatia; alajbeg@sfzg.unizg.hr; 4 Department of Dentistry, University Hospital Centre Zagreb, 10000 Zagreb, Croatia

**Keywords:** bruxism, sleep bruxism, signal detection theory, dental occlusion, tactile perception

## Abstract

**Objective**: This cross-sectional study investigated whether the frequency of waking-state and sleep-related bruxism is associated with sensitivity to occlusal tactile stimuli (i.e., occlusal tactile acuity) in healthy individuals. **Methods**: Forty healthy participants (20 females and 20 males) completed the Oral Behavioural Checklist to assess the frequency of waking-state and sleep-related bruxism. Participants were stratified into groups representing low, medium, and high bruxism frequency. Occlusal tactile acuity was evaluated using articulating foils of different thicknesses (8–56 μm) and sham trials presented in random order. Each stimulus was tested six times. Participants indicated whether they perceived a foil between their teeth. Signal detection theory was applied to distinguish perceptual sensitivity from response strategy. **Results**: Detection accuracy increased with foil thickness. Individuals with a high frequency of sleep-related bruxism exhibited reduced detection of thinner foils (8–24 μm) compared with those with low frequency of sleep-related bruxism (*p* = 0.029). Additionally, participants with high-frequency waking-state or sleep-related bruxism were more reluctant to report occlusal contact, indicating a more conservative reporting strategy (*p* = 0.045 and *p* = 0.002, respectively). **Conclusions**: Higher frequency of sleep-related bruxism was associated with reduced detection of low-intensity occlusal stimuli and a more conservative reporting strategy. These findings indicate an association between sleep-related bruxism frequency and differences in occlusal tactile detection. However, as the bruxism assessment was based on self-reports, these findings should be interpreted with caution.

## 1. Introduction

Bruxism is a repetitive oral motor behaviour characterised by grinding or clenching of the teeth, as well as by bracing or thrusting of the mandible, and it presents in two distinct circadian forms: waking-state bruxism (WB) and sleep-related bruxism (SB) [1]. Reported prevalence estimates for WB and SB vary widely across studies, largely due to differences in diagnostic methods [2,3,4]. Recent meta-analytic data indicate that both conditions are relatively common in the general population, although direct comparisons remain methodologically challenging [5]. The aetiology of bruxism is considered multifactorial, reflecting a complex interplay of genetic predisposition (e.g., polymorphisms in genes related to serotonergic and dopaminergic pathways), neurobiological mechanisms (e.g., alterations in GABAergic and other neurotransmitter systems), and psychological factors (e.g., stress and coping skills) [6,7,8]. Associations between obstructive sleep apnoea, sleep arousals, and sleep bruxism have been reported, although their nature remains complex and not fully understood [9]. No single mechanism fully explains bruxism. Bruxism is therefore best understood within a biopsychosocial framework, reflecting interactions between biological, psychological, and behavioural factors that modulate neural activity and may be expressed through orofacial parafunctional behaviours [10]. Occlusal factors such as malocclusion, once regarded as major aetiological contributors, lack strong evidence supporting a causal role in bruxism [11]. This multifactorial background complicates risk assessment and management. Among the clinical features most frequently associated with bruxism are tooth wear, restoration failures, and pain in the temporomandibular joint or masticatory muscles [12]. Because of its variable clinical presentation, no single sign or symptom is sufficient to confirm this oral parafunctional behaviour. Instead, assessment relies mainly on a combination of self-report and clinical findings. In addition, bruxism has been identified as a risk factor for temporomandibular disorders (TMD), particularly myofascial pain, and it is commonly assessed using instruments such as the Oral Behaviours Checklist (OBC), which is used within the behavioural framework of the Diagnostic Criteria for Temporomandibular Disorders (DC/TMD) [13,14]. The OBC is a validated questionnaire used to assess both functional and non-functional oral behaviours during wakefulness and sleep [15,16]. Within this instrument, six items specifically address non-functional behaviours, such as clenching, grinding, and sustained tooth contact, which are consistent with the current definition of WB [17]. Previous studies have shown that non-functional WB is associated with psychological distress, whereas functional oral activities during wakefulness do not appear to be similarly affected [18]. Alongside behavioural assessment, growing attention has been directed toward individual differences in tactile acuity, as these may also contribute to variability in bruxism-related behaviours. Sensitivity related to occlusal perception is commonly referred to as occlusal tactile acuity (OTA), defined as the ability to detect very small tactile differences between opposing teeth. According to the literature, observed differences in OTA among individuals may reflect not only sensory sensitivity but also cognitive and behavioural influences, such as attentional modulation and response bias [19,20]. Only a limited number of studies have investigated OTA in individuals with bruxism. One study found that individuals with either high or low frequencies of oral parafunctions differed in their ability to discriminate minimal tactile stimuli during biting in the intercuspal position [21,22]. Similarly, another study comparing individuals with SB and healthy controls reported differences in occlusal tactile thresholds, with SB participants showing lower thresholds [23]. In contrast, our previous study found an inverse relationship between the overall OBC score and occlusal sensitivity [24]. Taken together, these inconsistent findings suggest that the relationship between bruxism-related behaviours and occlusal sensitivity is complex and still not fully understood. Moreover, little is known about whether WB and SB show distinct relationships with OTA. These uncertainties highlight the need for approaches that can distinguish perceptual sensitivity from decision-related influences. Therefore, this study investigated whether the frequencies of waking-state and sleep-related bruxism are associated with OTA in healthy individuals, hypothesising that both sensory and behavioural factors influence responses to low-intensity stimuli. To further differentiate sensory profiles, signal detection theory was applied to dissociate perceptual sensitivity (d′) from decision strategy (c) [25]. A better understanding of this association may contribute to improved risk assessment and personalised management strategies for individuals with bruxism. However, it remains unclear whether these differences primarily reflect perceptual sensitivity or response-related processes.

## 2. Materials and Methods

### 2.1. Study Design

This cross-sectional study was conducted at the Department of Removable Prosthodontics, University of Zagreb School of Dental Medicine, and the Department of Dentistry, Clinical Hospital Centre Zagreb, Croatia. The study protocol followed the ethical principles of the Declaration of Helsinki and was reported in accordance with the STROBE guidelines [26,27] (Table S1). Ethical approval was obtained from the Ethics Committee of the School of Dental Medicine, University of Zagreb (05-PA-30-22-11/2023), and the trial was registered as NCT07163494 on ClinicalTrials.gov on 8 September 2025.

### 2.2. Study Participants

Data regarding bruxism frequency and occlusal sensitivity were collected between September and November 2025 from 40 healthy participants (20 females and 20 males). Participants were recruited from volunteers attending the University of Zagreb School of Dental Medicine and the University Hospital Centre Zagreb. All participants were of Southeast European ancestry. Written informed consent was obtained from all participants prior to inclusion in the study.

Inclusion criteria were age between 18 and 60 years, presence of natural dentition with occlusal contacts in all posterior supportive zones, and absence of missing premolars or molars at the test site. Exclusion criteria included the presence of prosthodontic or orthodontic appliances, current or previous history of TMD or other chronic orofacial pain conditions, periodontal disease, systemic diseases affecting neuromuscular or sensory function, and use of medication that could influence these functions.

A total of 56 volunteers were screened for eligibility. Sixteen individuals were excluded due to not meeting the inclusion criteria or declining participation, resulting in a final sample of 40 participants.

### 2.3. Bruxism Assessment

Bruxism frequency was assessed using the Croatian version of the Oral Behaviours Checklist (OBC), a validated 21-item self-report instrument designed to evaluate functional and non-functional oral behaviours during wakefulness and sleep [28].

The OBC consists of two domains: waking-state oral behaviours (19 items) and sleep-related oral behaviours (2 items). In the context of this study, waking-state oral behaviours were considered to reflect waking-state bruxism (WB), while sleep-related oral behaviours were considered to reflect sleep-related bruxism (SB). For the assessment of SB, the two sleep-related items (items 1 and 2) were used. WB was operationalised using six items representing non-functional behaviours such as clenching, grinding, or sustained tooth contact (items 3, 4, 5, 6, 7, and 11).

Each item is scored on a 5-point Likert scale ranging from 0 (“none of the time”) to 4 (“all of the time”). Composite scores were calculated as the sum of the relevant items. Higher scores indicated greater frequency of bruxism-related behaviours [15].

Based on the frequency of waking and sleep bruxism, subscores were categorised into three frequency levels, representing low, medium, and high tertiles [29].

### 2.4. OTA Assessment

Assessment of OTA was conducted upon completion of the OBC questionnaire. The procedure was conducted in an isolated and quiet room. Participants were blinded to visual and auditory input using an eye mask and noise-cancelling headset.

The assessment procedure followed our previously published protocol [24]. Seven articulating foils (Bausch, Köln, Germany) with thicknesses ranging from 8 μm to 56 μm in 8-μm increments, plus one sham trial without a foil, were applied to the right premolar/molar region. To minimise additional tactile stimuli, the buccal mucosa was gently retracted using a dental mirror. All foils were cut to a length of 2 cm. After foil placement, participants were instructed to close their mouths in a position where their teeth fit best. Each foil thickness was tested six times in a randomised order, resulting in 48 trials per participant. After each trial, participants reported whether they perceived a foil between their teeth (“yes” = 1, “no” = 0). For sham trials (no foil), correct responses were coded as “no” = 1 and incorrect responses as “yes” = 0.

Data were entered into a secure Google Forms database (Google LLC, Mountain View, CA, USA), where participant identifiers were coded numerically, and then exported to Microsoft Excel (Microsoft, Redmond, WA, USA) for statistical analysis.

The OTA assessment followed a previously standardised protocol to ensure procedural consistency. All stimuli were applied by a single trained examiner (IB), while a second researcher independently randomised trial order and recorded responses (IBo), thereby separating procedural roles. The examiner applying the foils was blinded to participants’ bruxism group allocation. The trial order was computer-randomised. Although formal intra-examiner reliability testing was not performed, the use of a single trained examiner helped ensure consistency of stimulus delivery across participants.

### 2.5. Sample Size Estimation

Sample size estimation was based on findings from a study comparing participants with high and low bruxism frequencies, which reported a 10.0% difference in the proportion of correct responses with a standard deviation of 8.0% [22]. For the present study, behavioural scores were analysed across three bruxism frequency groups (low, medium, and high). To adapt the calculation to this tertile-based design, a graded distribution of means was assumed, with the medium-frequency group positioned midway between the low- and high-frequency groups, corresponding to an expected difference of 5.0% between adjacent groups and 10.0% between the extreme groups.

Under these assumptions, the standardised effect size for one-way ANOVA was Cohen’s f = 0.51. With α = 0.05 and 80% power, the required total sample size was 41 participants. The final sample of 40 participants was therefore considered close to the required sample size and acceptable for tertile-based group comparisons within this study.

### 2.6. Statistical Analysis

All analyses were performed using IBM SPSS Statistics (version 29.0.2.0; IBM Corp., Armonk, NY, USA). Statistical significance was set at *p* < 0.05.

To examine whether bruxism frequency was associated with differences in occlusal tactile detection profiles in healthy participants, waking and sleep-related bruxism subscores were categorised into three frequency levels, representing low, medium, and high bruxism frequency. Tertile cut-off values were derived from the distribution of bruxism subscores within the study sample. This categorisation enabled the identification of behavioural phenotypes within a non-clinical population.

Detection performance (% correct responses) across foil thicknesses (8–56 μm and sham) was analysed using repeated-measures general linear models. Foil thickness was treated as a within-subject factor, while bruxism frequency groups (waking or sleep-related) served as the between-subject factor. Age and sex were included as covariates. When Mauchly’s test indicated violation of sphericity, Greenhouse–Geisser corrections were applied. Given the a priori hypothesis that behavioural modulation would preferentially affect low-intensity stimuli, the interaction between foil thickness and bruxism frequency groups was considered the primary outcome of interest. To assess the independent contributions of WB and SB, an additional model including both tertile variables simultaneously was performed. To assess potential multicollinearity between waking-state and sleep-related bruxism variables in the combined model, variance inflation factors (VIF) were calculated. VIF values > 5 were considered indicative of problematic multicollinearity.

Signal detection theory parameters were derived from hit and false alarm rates. Hit rate (HR) was defined as the proportion of “yes” responses when a foil was present, while false alarm rate (FAR) was defined as the proportion of “yes” responses during sham trials (no foil). A log-linear correction was applied to avoid boundary estimates:

HR = (Hits + 0.5)/(SignalTrials + 1),

FAR = (FalseAlarms + 0.5)/(NoiseTrials + 1).

Z-scores were obtained by transforming HR and FAR into standard normal deviates using the inverse cumulative distribution function.

The signal detection indices were then calculated as

d′ = Z(HR) − Z(FAR),

c = −0.5[Z(HR) + Z(FAR)]

Differences in perceptual sensitivity (d′) and response criterion (c) across bruxism frequency groups were examined using one-way ANOVA with Bonferroni-adjusted post hoc comparisons.

Effect sizes were reported as partial eta squared (η^2^) and interpreted as small (=0.01), medium (=0.06), and large (=0.14).

## 3. Results

### 3.1. Sample Description

The final analysis included 40 healthy participants, with a mean age of 37.27 ± 16.45 years, free of temporomandibular pain or clinical dysfunction. WB and SB subscores were categorised into three frequency levels (low, medium, and high) to enable comparison of behavioural profiles within the healthy cohort. For SB, participants were relatively evenly distributed across low (*n* = 15), medium (*n* = 15), and high (*n* = 10) frequency groups. In contrast, WB frequency groups were more unevenly distributed, with the majority of participants classified within the low-frequency group (*n* = 23), and fewer in the medium (n = 10) and high (*n* = 7) groups. A moderate positive correlation was observed between waking-state and sleep-related bruxism scores (r = 0.455, *p* = 0.003), indicating partial overlap between the two constructs. Variance inflation factors were low (VIF = 1.262), indicating no evidence of problematic multicollinearity.

### 3.2. Detection Performance Across Foil Thicknesses

Detection accuracy increased progressively with foil thickness, confirming the expected psychophysical gradient (main effect of thickness, Greenhouse–Geisser corrected *p* < 0.001; partial η^2^ ≈ 0.45).

**Figure 1:**
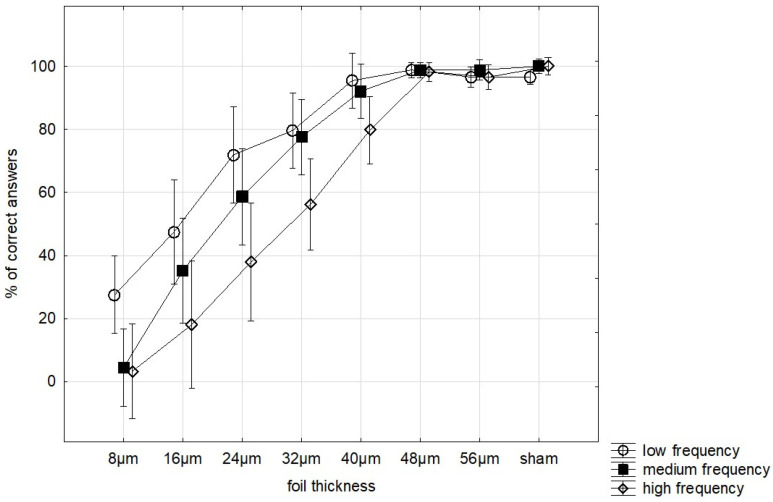
Detection accuracy across foil thickness by SB frequency groups. Vertical bars denote 0.95 confidence intervals.

When participants were stratified according to SB frequency, a significant interaction between foil thickness and SB frequency group was observed (Greenhouse–Geisser F = 1.99, *p* = 0.041, partial η^2^ = 0.142). Inspection of estimated marginal means demonstrated that individuals in the high-frequency SB group exhibited reduced detection performance at lower stimulus intensities (8–24 μm) compared with those in the low-frequency group. Differences between groups progressively diminished at higher thickness levels, with detection rates converging at ≥40 μm (Figure 1). This pattern suggests that the effect was primarily limited to low-intensity stimuli rather than reflecting a global shift in detection performance.

**Figure 2:**
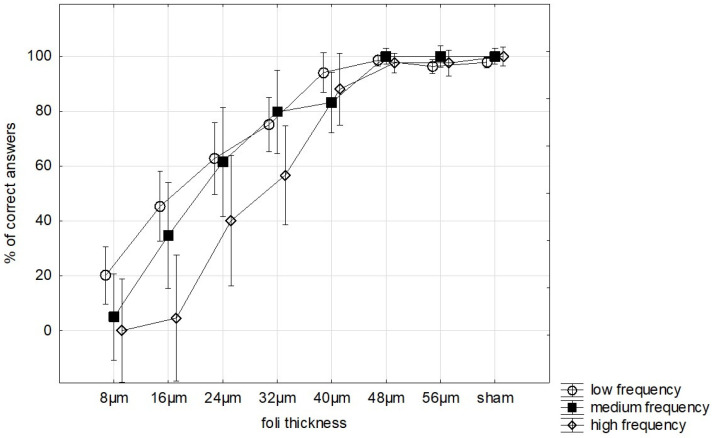
Detection accuracy across foil thickness by WB frequency groups. Vertical bars denote 0.95 confidence intervals.

When WB groups were analysed separately, a similar interaction between foil thickness and WB frequency group was observed (Greenhouse–Geisser F = 1.96, *p* = 0.042, partial η^2^ = 0.140) (Figure 2).

However, in the combined model including both WB and SB frequency groups simultaneously, only the interaction involving SB remained significant. The WB effect was no longer statistically significant, suggesting that the effects of waking-state and sleep-related bruxism may be partially interrelated (Table 1).

### 3.3. Signal Detection Parameters

To further distinguish perceptual sensitivity from response behaviour, signal detection parameters were analysed.

Analysis of perceptual sensitivity (d′) revealed significant differences across SB frequency groups (Table 2).

**Figure 3:**
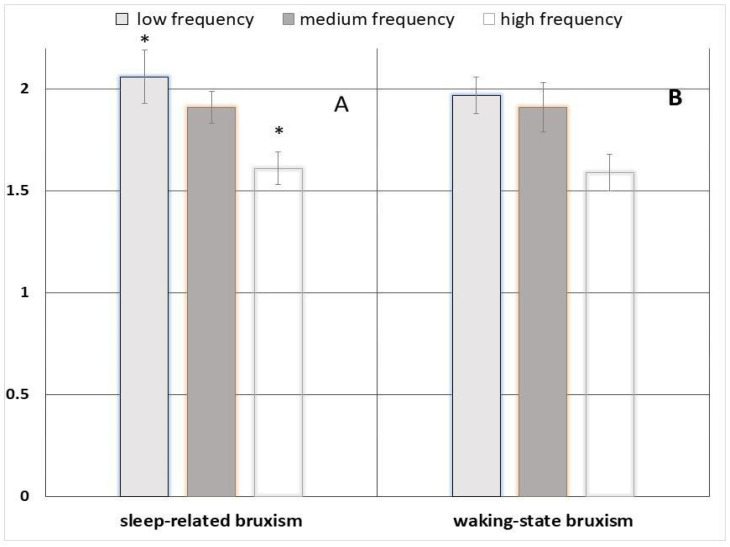
(**A**) Differences in perceptual sensitivity (d′) across SB frequency groups; (**B**) across WB frequency groups. * *p* < 0.05

Participants in the high-frequency SB group exhibited lower perceptual sensitivity (d′) compared with the low-frequency group (ANOVA F = 3.90, *p* = 0.029; Bonferroni-adjusted *p* = 0.025) (Figure 3A), indicating reduced ability to discriminate occlusal stimuli.

The response criterion (c), reflecting response bias, also differed significantly across SB frequency groups (F = 7.16, *p* = 0.002), with individuals in the high-frequency group adopting a more conservative decision strategy.

For WB, response criterion differed across frequency groups (F = 3.38, *p* = 0.045), with higher waking frequency associated with a more conservative criterion. In contrast, differences in perceptual sensitivity (d′) did not reach statistical significance (F = 2.43, *p* = 0.102) (Figure 3B), indicating that waking-state behaviours were not associated with measurable changes in perceptual sensitivity.

Overall, SB showed consistent associations with both detection accuracy and perceptual sensitivity, whereas WB was primarily related to response bias and did not demonstrate stable effects in the combined model.

## 4. Discussion

The main finding of this study was that sleep-related bruxism frequency was moderately associated with differences in occlusal tactile detection. Individuals with high-frequency SB exhibited reduced perceptual sensitivity (d′) and lower detection accuracy of thinner foils (8–24 μm) compared with those with low-frequency SB. Although high-frequency WB was also associated with reduced detection performance at lower stimulus intensities, only SB remained significant when both behaviours were included in the statistical model. A moderate correlation between WB and SB was observed (r = 0.455, *p* = 0.003), indicating that these constructs are partially related but not redundant. Additionally, multicollinearity diagnostics showed low variance inflation factors (VIF = 1.262), suggesting that both variables could be included in the model without introducing instability. However, despite the absence of problematic multicollinearity, their interrelationship may still complicate the interpretation of their independent contributions, and the findings should therefore be interpreted with caution. This pattern may indicate a more consistent association between SB frequency and occlusal tactile detection compared with WB within this sample. But, the lack of significance for WB may also reflect unmeasured heterogeneity in psychological characteristics within the study sample. Psychological factors may contribute to variability in occlusal tactile acuity, although these variables were not assessed in the present study [30]. Previous studies have reported associations between anxiety-related traits and reduced OTA, suggesting that attentional or cognitive factors may influence trial performance [31]. As such, the observed differences in OTA may not solely reflect sensory processing differences but also behavioural or psychological influences. Nevertheless, differences in OTA between individuals with different bruxism patterns appear to be evident and require further clarification. Differences between WB and SB are also reflected in clinical management strategies. While WB often responds well to behavioural modification therapy, the primary management strategy for SB is the use of a night guard to reduce tooth wear [32,33]. When the decision criterion was analysed, individuals with high-frequency WB and SB were more reluctant to report occlusal contact, indicating a more conservative decision strategy (c) compared with those with lower bruxism frequencies. These differences in trial performance reporting may reflect not only perceptual sensitivity but also variations in decision-making behaviour. Several mechanisms may account for this pattern [34,35]. Still, it remains unclear whether such effects are primarily peripheral, central, or behavioural in nature. The present findings, therefore, do not allow insight into underlying biological mechanisms. Rather, they support the notion that both sensory and non-sensory factors contribute to OTA performance. Regarding study limitations, the sample consisted of volunteers recruited from a single academic centre, which may introduce selection bias and limit the generalisability of the findings. As previously mentioned, psychological characteristics were not assessed, which may restrain the interpretation of these findings. Assessment of bruxism was based on self-reported instruments, which consist of only two questions for sleep-related behaviours, and self-report assessment of SB is known to have low sensitivity, representing a recognised limitation of questionnaire-based approaches [36]. Although other validated self-report instruments are available for assessing bruxism, the OBC was selected due to its reliability and ability to differentiate functional from non-functional oral behaviours [37,38]. Although the sample size was adequate for the planned analyses, larger samples would allow more precise estimation of subgroup differences. In addition, categorising continuous OBC scores into tertiles may have reduced statistical power and introduced artificial thresholds, potentially affecting the sensitivity of the analyses. Future studies should consider modelling bruxism frequency as a continuous variable. Although multicollinearity diagnostics indicated low variance inflation factors, the interrelationship between WB and SB may still limit the interpretation of their independent contributions. The uneven distribution of WB frequency groups, with a smaller number of participants in the high-frequency group, may have influenced statistical power and the stability of group comparisons. Although a standardised protocol and examiner blinding were used to minimise bias, formal intra-examiner reliability was not assessed, which may represent a methodological limitation. However, this study also has several important strengths. Our findings suggest that sensory profiles may differ depending on the frequency of parafunctional oral behaviours such as bruxism. The sample consisted of well-defined, healthy adults with natural dentition and stable occlusion. This provides insight into occlusal perception in a sample of healthy adults. Bruxism behaviours were assessed using a validated questionnaire. To further distinguish functional from non-functional oral behaviours related to WB, only bruxism-related OBC items (3, 4, 5, 6, 7, and 11) were included. OBC items assessing functional oral activities, such as yawning and chewing, may confound the assessment of bruxism-related behaviours. Additionally, signal detection theory was applied to distinguish sensory sensitivity from response strategy. These findings contribute to the understanding of occlusal perception in individuals with varying bruxism frequencies and highlight the importance of considering both sensory and behavioural factors in its assessment. Future multicentre studies with larger samples are needed to confirm these findings.

## 5. Conclusions

This study found that a higher frequency of self-reported sleep-related bruxism was associated with reduced sensitivity to low-intensity occlusal stimuli and a more conservative response strategy in healthy individuals. These findings suggest that differences in occlusal tactile detection may be related to the frequency of sleep-related bruxism, particularly at lower stimulus intensities. However, these findings should be interpreted with caution due to the cross-sectional design and the use of self-reported measures. Waking-state bruxism frequency was primarily associated with changes in response behaviour rather than perceptual sensitivity. Overall, the results highlight the importance of distinguishing between waking and sleep-related bruxism when evaluating occlusal tactile detection. Further research using larger samples and objective measures of bruxism is needed to confirm these findings and better understand the factors contributing to these differences.

## Figures and Tables

**Table 1:** Repeated-measures general linear model (GLM) analysis of detection accuracy: effects of foil thickness and bruxism frequency groups.

Effect	F	*p*	Partial η^2^
Foil thickness	29.74	<0.001	0.452
Foil thickness × SB frequency groups	1.99	0.041	0.142
Foil thickness × WB frequency groups	1.96	0.042	0.140
Combined model: foil thickness × SB frequency groups	1.99	0.041	0.142
Combined model: foil thickness × WB frequency groups	ns	ns	ns

ns—non-significant.

**Table 2:** Signal detection parameters (d′ and c) across SB frequency groups.

	Low (n = 15) Mean ± SD	Medium (n = 15) Mean ± SD	High (n = 10) Mean ± SD	F	*p*	Partial η^2^
perceptual sensitivity (d′)	2.06 ± 0.51	1.91 ± 0.32	1.61 ± 0.27	3.90	0.029	0.174
response criterion (c)	0.30 ± 0.33	0.51 ± 0.16	0.66 ± 0.13	7.16	0.002	0.279

## Data Availability

The data that support the findings of this study are available from the corresponding author upon reasonable request.

## Supplementary Materials

The following supporting information can be downloaded at: https://www.mdpi.com/article/10.3390/jcm15093469/s1, Table S1: STROBE Statement—Checklist designed to improve the transparency and quality of published cohort, case-control, and cross-sectional studies.
